# Rapid detection of human and animal respiratory viruses using Microbe Finder (MiFi^®^)

**DOI:** 10.3389/fmicb.2026.1743643

**Published:** 2026-02-25

**Authors:** Marcos R. Ribeiro-Junior, Kitty F. Cardwell, Daniele Nascimento, Andres S. Espindola, Akhilesh Ramachandran, Sushim K. Gupta, Donna Tyungu

**Affiliations:** 1 Institute for Biosecurity and Microbial Forensics, Oklahoma State University, Stillwater, OK, United States; 2 Department of Entomology and Plant Pathology, Oklahoma State University, Stillwater, OK, United States; 3 Microbe Finder, LLC (MiFi^®^), Stillwater, OK, United States; 4 Oklahoma Animal Disease Diagnostic Laboratory, College of Veterinary Medicine, Oklahoma State University, Stillwater, OK, United States; 5 Section of Pediatric Infectious Disease, Department of Pediatrics, University of Oklahoma Health Sciences Center, Oklahoma City, OK, United States

**Keywords:** bioinformatics pipeline, diagnostic specificity, e-probe diagnostic nucleic acid analysis, genomic surveillance, *in-silico* validation, pathogen detection, point-of-need diagnostics, viral genomics

## Abstract

Rapid and accurate detection of respiratory pathogens is essential for timely diagnosis, effective treatment, and outbreak monitoring in both human and veterinary medicine. We evaluated the Microbe Finder (MiFi^®^) software for detection of nine RNA viruses of human and veterinary clinical importance. Species specific signature sequences in the different pathogen genomes were identified, and specific electronic probe sets were curated using the MiFi^®^ software. Analytical specificity and sensitivity were evaluated through simulated metagenomes and public sequence databases, respectively. Host-specific internal control probes were designed to ensure diagnostic reliability and quality control. Diagnostic performance was assessed using Oxford Nanopore sequence data from clinical nasal swab samples. *In silico* validation showed 100% specificity across 83 datasets and limits of detection as low as 0.0010% of total reads (10 reads per 10^6^) for some targets. Internal controls generated stable background signals without interfering with pathogen detection. *In vivo* testing of 44 clinical samples matched PCR performance for Human respiratory syncytial virus (HRSV), Influenza B virus (IBV), Influenza A virus (IAV), Bovine respiratory syncytial virus (BRSV), and Canine distemper virus (CDV). These findings demonstrate that the MiFi^®^ software enables rapid, multiplex, and strain-specific detection of respiratory viruses in metagenomic sequence data without the need for advanced bioinformatics expertise. The approach supports scalable use in clinical laboratories, veterinary diagnostics for surveillance and triage, offering a valuable tool for improving respiratory pathogen detection across diverse settings.

## 1 Introduction

Respiratory pathogens remain a major cause of morbidity and mortality worldwide, affecting both humans and animals and imposing significant clinical and economic burdens. Effective surveillance systems are essential to monitor these infectious diseases, prevent outbreaks, and guide timely public health responses (World Health Organization, 2019). Among human diseases, the persistence of vaccine-preventable infections such as measles, mumps, and respiratory syncytial virus, driven by factors such as vaccine hesitancy, incomplete immunization, or waning immunity, highlights the need for improved diagnostic approaches (Dabbagh, 2018; Patel, 2020). Early symptoms of many childhood infections are difficult to diagnose due to how and when they manifest in patients, and substantial overlap in symptoms in early stages. Diseases for which differential diagnosis is confounded by many potential causal agents can result in delays in diagnosis, critical to effective containment and treatment. During public health surveillance programs, the ability to differentiate between geographic strains and vaccine strains is important. This study provides a proof of concept for novel diagnostic technology that has the potential to differentiate among any number of pathogens in the same sample and is specific to the strain level.

Traditional diagnostic methods, including culture-based techniques, ELISA, and PCR, have contributed greatly to pathogen detection but present notable limitations. Challenges include restricted multiplexing capability, longer turnaround times, and, in the case of PCR, limited resolution at the strain or genotype level (Mackay et al., 2002). Such constraints can delay or obscure accurate identification, thereby affecting epidemiological investigations and outbreak control.

High-throughput sequencing (HTS) has emerged as a powerful alternative, enabling the detection of multiple pathogens simultaneously and providing higher resolution for taxonomic classification (Chiu and Miller, 2019). Metagenomic sequencing of environmental samples has enabled the simultaneous detection of multiple human respiratory and enteric viruses directly from air, including under-monitored pathogens such as influenza C, demonstrating that, regardless of sample type, untargeted high-throughput or shotgun sequencing can detect and identify both expected and unexpected pathogens and strengthen outbreak detection and response (Minor et al., 2023). However, the routine integration of HTS into diagnostic workflows has been hindered by the need for specialized bioinformatics expertise, computational resources, and complex data interpretation (Wang et al., 2022).

The Microbe Finder (MiFi^®^) technology addresses these challenges by implementing e-probe-based diagnostics. E-probes are short, highly specific nucleotide sequences curated to detect targeted pathogens within HTS datasets. This approach offers rapid, accurate, and multiplex detection, while its user-friendly, web-based interface eliminates the need for advanced bioinformatics skills (Espindola and Cardwell, 2021). MiFi^®^ and E-probe Diagnostic Nucleic Acid Analysis (EDNA) methodologies have already demonstrated their versatility across diverse applications in plant pathology and veterinary diagnostics (Espindola et al., 2018; Bocsanczy et al., 2023; Dang et al., 2023; Narayanan et al., 2023; Proaño-Cuenca et al., 2023, 2025; Pasha et al., 2024; Nascimento et al., 2025; Peña-Zúñiga et al., 2025; Ribeiro-Junior et al., 2025; Roy et al., 2025).

This backdrop in R&D led to a collaborative project grant for the authors of this paper with the Centers for Disease Control and Prevention (CDC). The objective of the proof-of-concept project was to develop and validate e-probes for differential and strain-level diagnostics of RNA viruses from nucleic acid in nasal swabs. This study included valuative confirmation with known positive sample sequences provided by the CDC, and diagnostic samples from sequenced nasal swabs from Oklahoma University Children’s Hospital. Additionally, the pathogens of concern in human diagnostics have Genus-level counterparts in veterinary diagnostics, providing the opportunity to measure diagnostic specificity with known positive and negative veterinary nasal swab samples.

Results illustrate the potential to integrate multiplexed *in-silico* sequence analysis to differentiate respiratory pathogens in nasal swabs for routine disease diagnostics and to differentiate between closely related strains of RNA viruses for monitoring and tracking in public health surveillance. Our results show that MiFi^®^ e-probes in routine diagnostics can offer a rapid multiplex, accurate, and scalable solution for respiratory pathogen detection in both human and animal health contexts. Our longer-term vision and goal are to streamline pathogen detection and identification (to the strain level where relevant) in sequence data for practical pathology laboratory workflow, where bioinformatics is not available, to improve diagnostic testing and differentiation of causal agents of respiratory diseases.

## 2 Materials and methods

**FIGURE 1:**
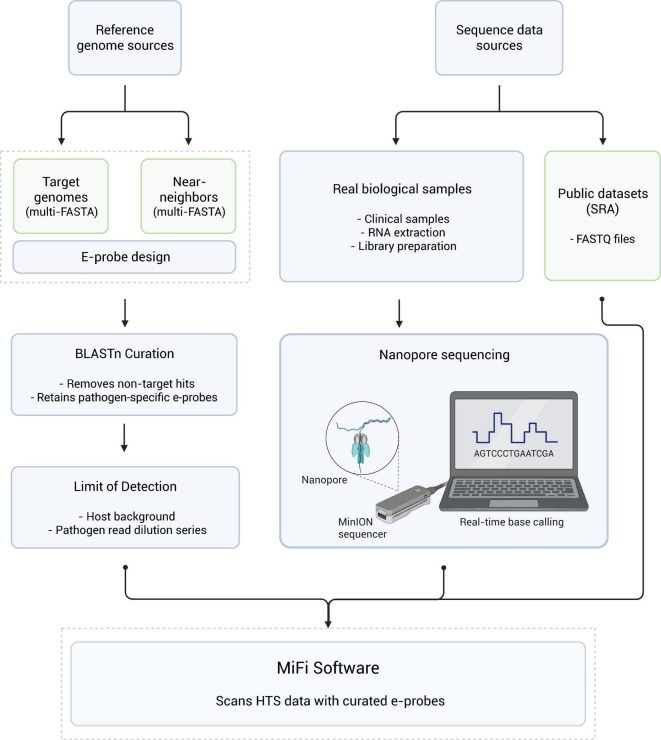
Overview of the MiFi^®^ pipeline for e-probe design, validation, and pathogen detection. Reference pathogen and near-neighbor genome sequences are used for in silico e-probe design and BLASTn-based curation, followed by limit-of-detection (LOD) assessment. Sequence data are obtained either from real biological samples sequenced using Nanopore technology or from publicly available SRA datasets. Curated e-probes are applied to high-throughput sequencing (HTS) data using the MiFi^®^ software. Dashed boxes indicate steps performed within the MiFi^®^ online software (bioinfo.okstate.edu and mifi.tech). Created with BioRender.com. BioRender publication and licensing rights were obtained under Agreement No. II29BDEBQ0.

We developed and validated e-probes for nine respiratory RNA viruses of human and veterinary importance using the MiFi^®^ pipeline (Figure 1). Human viruses included *Alphainfluenzavirus influenzae* (influenza A virus, IAV), *Betainfluenzavirus influenzae* (influenza B virus, IBV), *Morbillivirus hominis* (measles virus, MeV, including genotypes B3, D8, and vaccine strains Edmonston and Moraten), *Orthopneumovirus hominis* (human respiratory syncytial virus, HRSV), *Orthorubulavirus hominis* (human parainfluenza virus 4, HPIV4), and *Orthorubulavirus parotitidis* (mumps virus, MuV). Animal viruses included *Morbillivirus canis* (canine distemper virus, CDV), *Morbillivirus felis* (feline morbillivirus, FeMV), and *Orthopneumovirus bovis* (bovine respiratory syncytial virus, BRSV). The animal viruses were selected as genus-level representatives to provide validation to the genus-level when human virus samples were unavailable. We assessed their analytical specificity and sensitivity through *in silico* testing with mock sequence metagenomes and NCBI Sequence Read Archive (SRA) databases (Leinonen et al., 2010) and validated their diagnostic performance using Oxford Nanopore sequence data of clinical and veterinary nasal swab samples.

### 2.1 E-probe development and curation

Electronic probes (e-probes) were designed for each of the nine viruses using the MiFi^®^ probe developer pipeline, following the methods described by Espindola and Cardwell (2021). For MeV, e-probes were designed for a generic target, genotypes B3 and D8, and vaccine strains Edmonston (MeV-Edwt) and Moraten (MeV-Mor).

For each target virus, all available genome sequences belonging to the target taxon (inclusivity panel) were retrieved from NCBI and concatenated into a single multi-FASTA file. Closely related viruses and other relevant taxa expected to occur in similar sample matrices were compiled into a separate multi-FASTA file representing the near-neighbor (exclusivity) panel to ensure specificity during probe design (Supplementary Tables 1, 2).

The MiFi^®^ e-probe software^1^ generated raw e-probes by scanning target genomes for unique nucleotide regions absent from the near-neighbor dataset. E-probe length was selected based on sequence range recommended for viruses (20–60 nt) (Espindola and Cardwell, 2021). In this study, most e-probes were generated at 40 nt, whereas measles virus vaccine strains required shorter probes (20 nt for MeV-Edwt and 30 nt for MeV-Mor) to resolve closely related sequences. The total number of e-probes varied depending upon degree of sequence homology/differentiation with the nearest strains.

Candidate e-probes were initially selected based on sequence uniqueness and alignment characteristics, including percent identity and query coverage against near neighbors. Raw e-probes were subsequently subjected to a multi-step curation process to ensure high specificity and minimize the risk of false positives in downstream diagnostic applications.

Each e-probe set was queried against the NCBI GenBank nucleotide (nt) database using BLASTn to evaluate potential cross-reactivity. E-probes exhibiting ≥ 90% identity and ≥ 90% query coverage to non-target taxa, defined as organisms outside the intended virus NCBI taxonomy ID and its sub taxa, were classified as potentially cross-reactive and eliminated using a custom taxonomy-aware filtering script that parses BLASTn tabular output generated against the NCBI GenBank nucleotide (nt) database and evaluates each alignment based on percent identity, query coverage, and associated taxonomy identifiers (staxids). E-probes producing any high-confidence BLASTn hits to non-target taxonomic identifiers were excluded. For e-probes aligning to multiple taxa, retention was permitted only when all significant taxonomic assignments belonged to the target lineage; probes with any non-target taxonomic assignments were removed. This taxonomy-specific filtering step ensured retention of only highly specific e-probes uniquely associated with their respective viral targets and minimized the risk of false-positive detection.

The final curated e-probes were aligned against the full set of selected pathogen genomes to confirm exclusive association with their respective viral targets and to verify the absence of significant cross-alignment to other pathogens included in the study. This verification was performed using Geneious Prime software, providing an additional quality-control step to support accurate and reliable pathogen detection.

### 2.2 Internal control development and validation

Robust diagnostic workflows require internal controls to ensure assay integrity, particularly in HTS data where results depend on quantitative comparisons between positive and negative signals (Espindola and Cardwell, 2021; Wang et al., 2022). However, in highly specific e-probe sets, a challenge arises when negative controls exhibit zero signal variance. This can compromise downstream analytical interpretation and prevent the calculation of detection thresholds (*p*-values). To mitigate this, internal control (exogenous DNA or RNA) e-probes are introduced to generate a stable background signal in true-negative samples, ensuring that statistical metrics and detection criteria can be applied consistently across all datasets.

In this study, we implemented a dual internal control strategy, using host-specific internal control e-probes for human and animal samples. For use in human-derived clinical samples, an internal control e-probe ACTB_NM001101_40nt was designed from a highly conserved region of the *Homo sapiens* beta-actin (ACTB) gene (RefSeq accession NM_001101). This gene is abundantly expressed and stably present across diverse human tissues, making it a reliable endogenous control for clinical metagenomic workflows.

For use in animal samples, including bovine, canine, and feline specimens, a separate internal control e-probe, ACTB_NM173979_40nt, was designed from a conserved region of the *Bos taurus* ACTB gene (RefSeq accession NM_173979). This region was selected for its strong sequence conservation across multiple mammalian species, providing compatibility with different animal hosts while avoiding cross-reactivity with viral sequences.

To evaluate the performance and specificity of each internal control, we analyzed representative virus-free datasets: a publicly available human metagenomic dataset from the NCBI Sequence Read Archive (SRA accession SRR32080769) and an animal-derived negative dataset (SRA accession SRR33223349). Each internal control e-probe was integrated into the MiFi^®^ software alongside pathogen-specific e-probe databases, and background signal was quantified using *p*-values from e-probe alignment statistics.

### 2.3 Analytical sensitivity validation with mock metagenomic data

To simulate realistic host—pathogen metagenomic environments for *in-silico* sensitivity testing, HTS datasets free of target pathogens were selected as background. Specifically, *H. sapiens* sequencing data (SRA accession SRR32080769) was used to represent human genomic background, while *B. taurus* whole-genome sequencing data (SRA accession SRX28470509) was used for animal background simulations. Prior to use, both host datasets were analyzed using the full panel of curated e-probe sets to confirm that no false-positive signals were generated, ensuring their suitability as clean backgrounds for downstream mock metagenome construction.

For each pathogen, reference nucleotide sequences were retrieved from GenBank (accession numbers listed in Supplementary Table 3) and used as input for read simulation. Simulated reads were generated using ART Illumina (version 2.5.8, released June 6, 2016) with the built-in HiSeq 2500 profile (-ss HS25), producing 150 bp paired-end reads (–p –l 150) with an average fragment length of 300 bp and a standard deviation of 10 bp (–m 300 –s 10). A total of 10,000 paired-end reads were simulated for each reference sequence.

The simulated pathogen reads were randomly subsampled to create spike-in datasets at defined concentrations, ranging from 1 to 130 reads per million (RPM). For each mock metagenome, the total number of reads was fixed at 1 million. To achieve this, the appropriate number of pathogen reads was combined with host-derived reads from the selected SRA datasets, ensuring that the total read count remained constant while the proportion of pathogen reads varied according to the target concentration.

The spike-in and dataset assembly processes were implemented using a custom Python script, which utilized the pysam, Biopython, argparse, and random libraries for file manipulation, parsing, and reproducible sampling. For each concentration level, 10 independent replicates were generated to enable statistical assessment of detection thresholds.

Each resulting simulated metagenome was uploaded to the MiFi^®^ detection software^2^ as a “Metagenome” file and analyzed independently using the corresponding curated e-probe sets. Detection was performed using appropriate e-probe library selections, with default parameters for viral targets, specifically an e-value threshold of 1e^–1^ and a minimum of 10 sensitive hits, as recommended by the software developers (Espindola and Cardwell, 2021). A sample was considered positive for a given pathogen when the calculated was ≤ 0.05. The limit of detection (LoD) for each e-probe set was defined as the lowest concentration at which at least 9 out of 10 replicates yielded positive detection.

This approach enabled a biologically relevant *in-silico* evaluation of probe performance under simple metagenomic conditions.

### 2.4 Additional *in-silico* diagnostic validation of e-probes using NCBI sequence read archive datasets

For more complex *in-silico* validation, publicly available high-throughput sequencing datasets from the NCBI SRA were selected based on confirmed infection with specific target pathogens. Each dataset was uploaded to the MiFi^®^ software and analyzed with the complete panel of curated e-probe sets. Only the set corresponding to the pathogen confirmed by prior testing was expected to yield a positive result, while all others served as negative controls. Diagnostic outputs, including the calculated and the final classification result (positive or negative), were recorded for each e-probe set. Any e-probe sequence that produced false-positive results in non-target datasets were identified and removed from the final diagnostic sets to improve specificity.

### 2.5 Diagnostic sensitivity validation on clinical samples and Oxford Nanopore technologies

To evaluate the diagnostic sensitivity of MiFi^®^ virus-specific e-probes, a total of 44 respiratory samples, 16 pediatric human nasal swabs and 28 known-status animal samples (BRSV and CDV; nasal swabs and brain/lung tissue), were processed using a standardized Oxford Nanopore Technology (ONT) RNA sequencing workflow. Clinical samples were provided by the Oklahoma Children’s Hospital at OU Health and processed in a university research laboratory in Oklahoma City, OK, USA and animal samples were processed at Oklahoma Animal Disease Diagnostic Laboratory at Oklahoma State University in Stillwater, OK, USA.

Nasal swab samples were collected and stored in BD universal viral transport (UVT) (BD, Cat. No. 220526). Total RNA was extracted from 200–500 μL of each sample using the *PureLink Viral RNA/DNA Mini Kit* (Thermo Fisher Scientific, Cat. No. 12280050) according to the manufacturer’s instructions. This included proteinase K digestion, lysis with carrier RNA, and purification via silica spin columns. Elution was performed in 50 μL of RNase-free water. RNA concentration was quantified with the Qubit^®^ RNA HS Assay Kit (Invitrogen, Cat. No. 10320093).

To reduce host ribosomal background, 1–5 μg of total RNA per sample underwent rRNA depletion using the *RiboMinus Eukaryote System v2* (Thermo Fisher Scientific, Cat. No. A15026). Biotinylated probes targeting cytoplasmic and mitochondrial rRNA were hybridized to the RNA and removed via streptavidin-coated magnetic beads. rRNA-depleted RNA was then purified using the system’s magnetic bead clean-up module and eluted in 12 μL of 70°C nuclease-free water.

Because the target viruses lack natural polyadenylation and the ONT library preparation protocol required poly(A) tails, we performed 3’ polyadenylation using *E. coli* Poly(A) Polymerase (NEB, Cat. No. M0276L) following Oxford Nanopore’s recommended protocol. Briefly, 1–10 μg of rRNA-depleted RNA was incubated with PAP, ATP, and reaction buffer at 37°C for 1 min to append 50–100 adenosine residues. The reaction was stopped with 50 mM EDTA, and the RNA was purified using RNAClean XP beads (Beckman Coulter, Cat. No. A63987), then resuspended in 12 μL of nuclease-free water for downstream use.

Polyadenylated RNA was used as input for the *Oxford Nanopore cDNA-PCR Sequencing Kit V14 with barcoding* (SQK-PCB114.24), following the manufacturer’s instructions. First, reverse transcription and strand-switching were performed using CRTA adapters with poly(T) overhangs to ensure capture of full-length polyadenylated transcripts. Second-strand synthesis was followed by 18 cycles of PCR amplification using barcoded primers. Libraries were cleaned with AMPure XP beads, quantified using the Qubit^®^ fluorometer.

Equimolar pooling of barcoded libraries was performed to a final concentration of 50 fmol in 11 μL for adapter ligation. The pooled library was sequenced on a *MinION Mk1B device* (ONT) using R10.4.1 flow cells (FLO-MIN114). Sequencing was conducted using *MinKNOW software*, and high accuracy base calling was performed in real time with *Dorado*, the ONT base calling software.

The Nanopore metatranscriptomic datasets were uploaded to the MiFi^®^ software, which automatically screened the reads using e-probes selected from the CDC library of e-probes, following the recommended parameters for virus detection (Espindola and Cardwell, 2021). Screening was performed by aligning sequencing reads against the e-probe database. Positive matches indicated the presence of target viral sequences in the dataset. The E-value threshold was set at 1e–1, and the sensitivity level required a minimum of 10 matching reads to report a positive detection.

For confirmation, metatranscriptomic ONT reads were quality-trimmed with fastp. Host reads were removed by aligning to the appropriate host reference genome(s) with minimap2 (preset map-ont) and retaining the unmapped reads. The host-depleted reads were then aligned to the pathogen reference genomes listed in Supplementary Table 3 using minimap2. For each target virus, we quantified the number of mapped reads per reference and the genome breadth of coverage (%), defined as the percentage of reference bases with ≥ 1 × coverage.

Sequence data were analyzed and compared with PCR confirmation previously provided by the diagnostic clinic(s), as well as with the outputs from the MiFi^®^ software. Diagnostic sensitivity was calculated by comparing these results using the online calculator developed by Groth-Helms et al. (2025) and available through the Diagnostic Assay Validation Network (DAVN) website.^3^

## 3 Results

### 3.1 E-probe development and curation

The MiFi^®^ e-probe development process produced 13 distinct probe sets tailored to the genomic characteristics and diagnostic requirements of 9 respiratory pathogens (Table 1). For human viruses, most e-probes were 40 nucleotides in length, although the measles virus strain probe sets required a more targeted sensitivity (smaller e-probe lengths) to differentiate closely related vaccine strains. The generic measles virus set, built from all publicly available MeV sequences in GenBank, yielded 2042 e-probes capable of detecting diverse genotypes. In contrast, the vaccine strains Edmonston (MeV-Edwt) and Moraten (MeV-Mor) resulted in much smaller and highly specific probe sets, 2 and 11 e-probes, respectively, due to the low genetic variability within these strains and the limited sequence availability. For example, only one genome was available for MeV-Mor. Six highly similar near neighbors were included in the design, restricting the number of unique regions available for probe development. Additionally, these probes were shorter (20 nt for Edwt and 30 nt for Mor) because the conserved vaccine regions offered few mutations and required high-resolution targeting.

**TABLE 1:** Target pathogens, e-probe set, e-probe length, and number of e-probes.

Target pathogen	Acronym	E-probe set	E-probe length (nt)	No. of e-probes
**Human viruses**				
Human parainfluenza virus 4	HPIV4	HPIV4_40nt_v10	40	45
Human respiratory syncytial virus	HRSV	HRSV_40nt_v13	40	71
Influenza A virus	IAV	IAV_40nt_v3	40	4,663
Influenza B virus	IBV	IBV_40nt_v3	40	1,014
Measles virus (generic set)	MeV	MeV_40nt_v3	40	2,042
Measles virus B3	MeV-B3	MeV-B3_40nt_v10	40	12
Measles virus D8	MeV-D8	MeV-D8_40nt_v10	40	26
Measles virus Edwt*	MeV-Edwt	Edwt_20nt_v2	20	2
Measles virus Mor*	MeV-Mor	Mor_30nt_v2	30	11
Mumps virus	MuV	MuV_40nt_v10	40	1,183
**Animal viruses**				
Bovine respiratory syncytial virus	BRSV	BRSV_40nt_v10	40	83
Canine distemper virus	CDV	CDV_40nt_v10	40	436
Feline morbillivirus	FeMV	FeMV_40nt_v10	40	522

HPIV4, human parainfluenza virus 4; HRSV, human respiratory syncytial virus; IAV, influenza A virus; IBV, influenza B virus; MeV, measles virus; MuV, mumps virus; BRSV, bovine respiratory syncytial virus; CDV, canine distemper virus; FeMV, feline morbillivirus.

*Edwt, Edmonston wild-type strain; Mor, Moraten strain.

A similar pattern was observed for the IAV probe set, which generated 4,663 e-probes, the highest among all targets, driven by the vast number of sequences available in GenBank (over 1 million entries). This contrast illustrates how the volume of available genomic data strongly influences probe diversity: targets with high sequence representation and variability generate large, inclusive probe sets, whereas narrowly defined, highly conserved targets with minimal data yield fewer, more specific probes, sacrificing sensitivity, thus potentially requiring deeper sequencing to detect.

### 3.2 Internal control development and validation

The internal control e-probe ACTB_NM001101_40nt, derived from the *H. sapiens* ACTB gene, was validated using a virus-free human metagenomic dataset (Table 2). The probe consistently generated background signal without interfering with pathogen-specific alignments, confirming its suitability for distinguishing true-negative results in human-derived samples.

**TABLE 2:** Performance of pathogen-specific e-probe sets and internal controls in a virus-free metagenomic dataset (SRR32080769).

Target pathogen	Acronym	*p*-value	Diagnostic result
**Human viruses**			
Human parainfluenza virus 4	HPIV4	1.64E-01	Negative
Human respiratory syncytial virus	HRSV	1.62E-01	Negative
Influenza A virus	IAV	1.59E-01	Negative
Influenza B virus	IBV	1.59E-01	Negative
Measles virus (generic set)	MeV	1.59E-01	Negative
Measles virus B3	MeV-B3	1.77E-01	Negative
Measles virus D8	MeV-D8	1.67E-01	Negative
Measles virus Edwt*	MeV-Edwt	1.67E-01	Negative
Measles virus Mor*	MeV-Mor	1.27E-01	Negative
Mumps virus	MuV	1.59E-01	Negative
**Animal viruses**			
Bovine respiratory syncytial virus	BRSV	1.62E-01	Negative
Canine distemper virus	CDV	1.59E-01	Negative
Feline morbillivirus	FeMV	1.59E-01	Negative

Internal controls: ACTB_NM001101_40nt was used for human e-probes; ACTB_NM173979_40nt for animal e-probes.

*Edwt, Edmonston wild-type strain; Mor, Moraten strain.

For animal pathogens, the internal control e-probe ACTB_NM173979_40nt, designed from a conserved region of the *B. taurus* ACTB gene, was validated using whole-genome sequencing data from bovine samples. The probe similarly produced consistent, background signals across animal-derived datasets, demonstrating reliable performance as an internal control for bovine, canine, and feline samples (Table 2).

These results confirm that both host-specific internal controls provide stable, low-level signals that support robust discrimination between true-negative and low-titer samples without cross-reactivity detection events. All pathogen-specific e-probe tests returned negative results when tested against pathogen-free host HTS data, confirming the absence of cross-reactivity and validating the specificity of the internal control strategy (Table 2). The observed *p*-values ranged from 0.127 to 0.177, reflecting consistent background signal generation without interference in pathogen detection.

The background signal introduced by the internal control provided consistent normalization across the tested datasets, ensuring robust pathogen detection in the absence of confounding factors.

### 3.3 Analytical sensitivity validation with mock metagenomic data

*In silico* analyses using simulated metagenomes revealed that the theoretical limit of detection (LoD) varied across the curated e-probe sets (Table 3). LoD is defined as the minimum number of target reads detected within a simulated pool of 1 × 10^6^ total reads and are expressed as a percentage of total reads.

**TABLE 3:** Analytical sensitivity (limit of detection, LoD) of e-probe sets in simulated metagenomic datasets, reported as percentage of total reads.

Target pathogen	Acronym	LoD	
		% of total reads	Reads per 10^6^
**Human viruses**			
Human parainfluenza virus 4	HPIV4	0.0130	130
Human respiratory syncytial virus	HRSV	0.0130	130
Influenza A virus	IAV	0.0010	10
Influenza B virus	IBV	0.0010	10
Measles virus (generic set)	MeV	0.0010	10
Measles virus B3	MeV-B3	0.0130	130
Measles virus D8	MeV-D8	0.0100	100
Mumps virus	MuV	0.0010	10
**Animal viruses**			
Bovine respiratory syncytial virus	BRSV	0.0025	25
Canine distemper virus	CDV	0.0050	50
Feline morbillivirus	FeMV	0.0020	20

LoD values were determined *in silico* as the minimum number of target reads detected within a simulated pool of 1 × 10^6^ total reads and are expressed as a percentage of total reads. Conversion: percentage = (reads per 10^6^/10^6^) × 100 = (reads per 10^6^) × 0.0001.

Among human viruses, the e-probes targeting IAV, IBV, MuV, and the generic MeV set showed the highest sensitivity, each detecting as few as 0.0010% of total reads (equivalent to 10 reads per 10^6^). This is consistent with higher e-probe numbers per target. By contrast, the MeV-D8 genotype required 0.0100% (100 reads per 10^6^) to reach the same detection threshold, while MeV-B3, HPIV4, and HRSV e-probes had LoDs of 0.0130 (130 reads per 10^6^). The vaccine strain MeV-Edwt was represented by only two e-probes and therefore could not be assigned a definitive LoD under the criteria used in this study.

For animal viruses, the e-probe sets targeting BRSV, CDV, and FeMV exhibited LoDs of 0.0025, 0.0050, and 0.0020% of total reads, respectively (equivalent to 25, 50 and 20 reads per 10^6^) (Table 3).

### 3.4 *In-silico* validation of e-probes using NCBI sequence read archive datasets

The *in-silico* validation results showed that all e-probe sets successfully detected their corresponding target pathogens across the tested datasets on the MiFi^®^ software (Table 4). All samples were classified as positive, indicating that each e-probe set was able to detect its target pathogen with high confidence. These results support the sensitivity and effectiveness of the designed e-probes across a diverse panel of sequencing datasets. Notably, datasets for HRSV and MeV-D8 included sequences generated by both Oxford Nanopore and Illumina platforms, while all other targets were validated using Illumina-derived datasets only. This further highlights the robustness of the e-probes across different sequencing technologies. As expected, only the e-probe set corresponding to the confirmed pathogen yielded a positive signal, while all non-target e-probe sets remained negative, indicating no detectable cross-reactivity.

**TABLE 4:** *In-silico* validation of e-probes using NCBI sequence read archive (SRA) datasets.

Target pathogen (E-probe set)	SRA accession no.	Sequencing platform	EDNA-MiFi results	
			*p*-value	Diagnostic result
Measles virus D8 (MeV-D8_40nt_v10)	SRR31023696	Nanopore	1.35E-07	Positive
	SRR31023628		4.72E-07	Positive
	SRR31023629		6.56E-08	Positive
	SRR31023630		7.05E-08	Positive
	SRR31023631		4.23E-07	Positive
Measles virus B3 (MeV-B3_40nt_v10)	SRR25426228	Illumina	1.64E-04	Positive
	SRR25426231		3.70E-05	Positive
	SRR25426233		1.64E-04	Positive
	SRR25426229		1.64E-04	Positive
	SRR25426230		3.71E-05	Positive
Measles virus Edwt (MeV-Edwt_42nt_v2)	ERX2403188	Illumina	2.92E-02	Positive*
Canine distemper virus (CDV_40nt_v10)	SRR29428511	Illumina	7.04E-175	Positive
	SRR29428512		2.75E-198	Positive
	SRR23381486		1.01E-68	Positive
	SRR29428510		6.84E-196	Positive
	SRR23381487		1.77E-86	Positive
Feline morbillivirus (FeMV_40nt_v10)	SRR24829513	Illumina	2.02E-158	Positive
Human parainfluenza virus 4 (HPIV4_40nt_v10)	SRR19134725	Illumina	5.17E-04	Positive
	SRR19554363		5.09E-14	Positive
	SRR31796803		2.48E-11	Positive
	SRR31796802		5.09E-14	Positive
	SRR31796810		5.17E-04	Positive
Human respiratory syncytial virus (HRSV_40nt_v13)	SRR31926597	Illumina	3.00E-08	Positive
	SRR31926596	Nanopore	1.10E-08	Positive
	SRR31760625		3.01E-10	Positive
	SRR31980217		1.22E-08	Positive
	SRR31980221		2.06E-08	Positive
Mumps virus (MuV_40nt_v10)	SRR31089966	Illumina	*p* < 1E-308	Positive
	SRR31089967		*p* < 1E-308	Positive
	DRR453829		4.39E-51	Positive
	SRR26820343		*p* < 1E-308	Positive
	SRR24739250		2.21E-282	Positive
Bovine respiratory syncytial virus (BRSV_40nt_v10)	SRR22019715	Illumina	3.86E-173	Positive
	SRR22019749		6.88E-115	Positive
	SRR22019722		3.64E-172	Positive
	SRR22019727		1.25E-11	Positive
Influenza B virus (IBV_40nt_v3)	SRR33020280	Illumina	3.03E-209	Positive
	SRR33020281		1.91E-89	Positive
	SRR33020282		7.13E-211	Positive
	SRR33020283		3.19E-185	Positive
	SRR33020284		4.26E-270	Positive
	SRR32966402		2.54E-282	Positive
	SRR33011819		1.49E-319	Positive
Influenza A virus (IAV_40nt_v3)	SRR33018984	Illumina	1.87E-145	Positive
	SRR33018985		1.05E-144	Positive
	SRR33018986		8.59E-73	Positive
	SRR33018987		2.90E-71	Positive
	SRR33018988		1.81E-126	Positive
	SRR33020020		9.22E-153	Positive
	SRR33020021		1.90E-164	Positive
	SRR33020022		1.06E-95	Positive
	SRR33020023		4.68E-194	Positive
	SRR33020024		7.26E-103	Positive
	SRR33011823		7.58E-173	Positive
	SRR33011824		2.18E-188	Positive
	SRR33011825		1.99E-176	Positive
	SRR33011826		2.94E-185	Positive
	SRR33011827		2.62E-226	Positive

*Positive call at an extremely sensitive threshold: minimum number of hits set to 1.

### 3.5 Diagnostic sensitivity validation on clinical samples using Oxford Nanopore technologies

A total of 44 human and animal clinical samples (PII data-disaggregated and with known pathology status) were initially selected for the detection of BRSV, CDV, IAV, IBV, and HRSV using Nanopore HTS in combination with the MiFi^®^ software and virus-specific e-probes. MiFi^®^ detection of the correct pathogen across all targeted viruses, showed strong concordance with PCR results. MiFi^®^ matched the performance of PCR in detecting viral pathogens, demonstrating performance comparable to PCR across the targets evaluated (Table 5).

**TABLE 5:** Comparative detection of respiratory viruses in clinical samples using PCR, high-throughput sequencing (HTS), and the MiFi^®^ software.

Pathogen	PCR result	HTS analysis (RefSeq), %	EDNA-MiFi		
			MiFi result	*p*-value*	E-probe set
BRSV	(+)	9.1	(+)	2.00E-03	BRSV_40nt_v1.fasta
	(+)	7.2	(+)	2.27E-02	
	(+)	14	(+)	8.08E-09	
	(+)	46.8	(+)	2.93E-14	
	(+)	0.9	(+)	2.27E-02	
	(+)	1.0	(+)	2.27E-02	
	(+)	6.2	(+)	2.20E-03	
	(–)	13.4	(+)	1.55E-05	
	(+)	10.7	(+)	1.00E-04	
	(–)	32.2	(+)	5.82E-10	
	(+)	57.4	(+)	2.13E-19	
	(+)	14.6	(+)	3.89E-07	
	(+)	6.9	(+)	5.90E-03	
CDV	(–)	24.2	(+)	7.68E-14	CDV_40nt_v10.fasta
	(+)	9.3	(+)	4.63E-02	
	(+)	94.0	(+)	5.63E-65	
	(+)	64.6	(+)	2.96E-28	
	(+)	100	(+)	9.07E-54	
	(–)	17.7	(+)	3.16E-14	
	(–)	23.4	(+)	7.29E-15	
	(+)	9.8	(+)	7.29E-15	
	(+)	100	(+)	3.66E-66	
	(–)	60.4	(+)	6.63E-33	
	(+)	67.3	(+)	1.40E-30	
	(+)	96.0	(+)	8.63E-37	
	(+)	100	(+)	1.28E-47	
	(+)	77.6	(+)	1.28E-47	
	(+)	31.8	(+)	1.28E-47	
HRSV	(+)	75.9	(+)	1.00E-04	HRSV_40nt_v14.fasta
	(+)	100	(+)	3.90E-03	
	(+)	100	(+)	2.38E-04	
	(+)	28.5	(+)	1.83E-02	
	(+)	61.2	(+)	4.75E-05	
	(+)	12.6	(+)	4.75E-05	
	(+)	8.8	(+)	2.05E-02	
	(+)	86.0	(+)	1.21E-02	
	(+)	47.0	(+)	2.50E-03	
IAV	(+)	23.5	(+)	1.88E-24	IAV_40nt_v3.fasta
	(+)	61.8	(+)	2.16E-22	
	(+)	35.4	(+)	1.54E-49	
	(+)	15.0	(+)	1.22E-25	
	(+)	10.7	(+)	2.27E-02	
IBV	(+)	94.1	(+)	2.27E-02	IBV_40nt_v3.fasta
	(+)	40.9	(+)	1.00E-04	

*MiFi^®^ *p*-values represent the statistical significance of e-probe hit enrichment relative to background signal, as calculated by the MiFi^®^ platform. Samples were considered MiFi^®^ positive when *p* ≤ 0.05.

MiFi^®^ identified several additional positives that were not detected by PCR for BRSV and CDV. While these may reflect low-abundance viral sequences not detected by PCR, the possibility of contamination or read carryover cannot be excluded. All MiFi^®^ positive detections were supported by statistically significant *P*-values and HTS RefSeq mapping results, further validating the analytical robustness of the software.

### 3.6 Bovine respiratory syncytial virus

Of the 13 samples that tested positive for BRSV by PCR, MiFi^®^ successfully detected the virus in all cases, resulting in a complete concordance with PCR (Table 5). This included samples with low viral abundance based on HTS analysis (e.g., 0.9 and 1.0%). In addition, MiFi^®^ (and mapping to reference analysis) detected BRSV in two PCR-negative samples, suggesting potential false negatives in PCR testing, or alternatively, the possibility of contamination or read carryover during the sequencing process.

### 3.7 Canine distemper virus detection

MiFi^®^ detected CDV in all 11 PCR-positive samples, as well as in four PCR-negative samples, yielding a diagnostic sensitivity of 100% (Table 5). These additional detections may indicate false negatives by PCR, or alternatively, contamination or read carryover during the sequencing process. Notably, MiFi^®^ consistently identified CDV even in samples with low RefSeq mapping percentages (e.g., 9.3 and 9.8%).

### 3.8 Human respiratory syncytial virus detection

The MiFi^®^ software identified HRSV in all nine PCR-positive samples, with genome coverage percentages from RefSeq mapping ranging from low (8.8%) to complete (100%) (Table 5). No false positives or false negatives were observed, indicating a diagnostic sensitivity of 100%.

### 3.9 Influenza A virus and influenza B virus detection

MiFi^®^ detected IAV in five PCR-positive samples and IBV in two PCR-positive samples (Table 5); diagnostic sensitivity was 100% for both viruses.

## 4 Discussion

This study aimed to develop and validate a set of e-probes for the metagenomic detection of nine respiratory viruses of clinical and veterinary importance using the MiFi^®^ software. The results demonstrated high *in-silico* specificity (100% across 83 datasets) and low limits of detection for most targets. The e-probes for BRSV, CDV, IAV, IBV, and HRSV were validated using real clinical samples, demonstrating 100% diagnostic sensitivity for all targets.

Miah et al. (2023) noted that viral abundance in clinical material depends on disease stage and severity and can be further masked by high levels of host nucleic acids. To address this, they implemented DNase I treatment to reduce host DNA and applied a two-step multiplex PCR to enrich all genome segments of IAV prior to sequencing. This targeted enrichment was feasible because they were working with a single, pre-identified pathogen. In contrast, our goal is to screen blind samples for multiple potential targets simultaneously; performing separate enrichment reactions for nine pathogens would greatly increase turnaround time and undermine the purpose of using an untargeted NGS approach. In this proof-of-concept study, we tested viruses that were predetermined and known to lack natural poly(A) tails (required for the library preparation workflow). In such cases, we used a single, simple enzymatic polyadenylation step prior to sequencing on the ONT platform, enabling direct, untargeted analysis of the sample in MiFi^®^ against the full panel of possible targets.

For BRSV and CDV, the MiFi^®^ software detected viral presence in samples that were negative by PCR but showed substantial genome coverage when mapped to reference sequences during HTS analysis.

The performance of the e-probes reflected the influence of sequence database size and diversity. Targets with broad genomic representation and high variability, such as IAV and generic MeV, generated larger and more sensitive probe sets, achieving limits of detection as low as 0.0010% (10 reads per 10^6^). In contrast, genotypes with limited sequence availability and high similarity to near neighbors, such as MeV-B3 and HPIV4, exhibited higher detection thresholds. This outcome is consistent with previous reports indicating that both database diversity and the availability of genomic regions that are clearly distinct from non-targets are critical determinants of probe sensitivity and specificity (Stobbe et al., 2013; Proaño-Cuenca et al., 2025). These observations highlight the need for comprehensive, regularly updated reference databases to optimize e-probe design and diagnostic performance.

This study also reinforces the utility of computational validation prior to laboratory implementation. The bioinformatics-driven approach enabled rapid assessment of probe specificity and sensitivity, minimizing the need for extensive wet-lab screening in the initial phases. Such *in silico* benchmarking aligns with the approach proposed by Espindola (2024), who highlights the value of standardized artificial HTS datasets for objectively assessing diagnostic performance metrics before empirical testing.

The implementation of host-specific internal controls, targeting conserved ACTB gene regions in *H. sapiens* and *B. taurus*, was critical for ensuring the reliability of negative results. These controls consistently generated stable background signals without interfering with pathogen-specific alignments, enabling the distinction between truly negative samples and those potentially affected by technical failures or poor sequencing quality.

Compared with traditional diagnostic methods, the MiFi^®^ software offers advantages that directly address common barriers to HTS adoption in diagnostics (Espindola and Cardwell, 2021). By enabling rapid interpretation of complex sequencing data without advanced bioinformatics expertise, supporting multiplex pathogen detection, and integrating seamlessly with portable sequencing devices such as the Oxford Nanopore MinION, MiFi^®^ increases the feasibility of deploying metagenomic diagnostics in routine laboratory workflows, in field-based investigations, and in resource-limited regions. The combination of speed, accessibility, and broad target coverage positions MiFi^®^ as a promising tool for syndromic surveillance, outbreak control, and early detection of zoonotic threats.

Nanopore MinION-based sequencing has already been successfully implemented in some clinical laboratories, where it enables comprehensive pathogen identification directly from patient samples, often delivering results in up to 24 h (Miah et al., 2023; Williams et al., 2023; Cane et al., 2024). In these examples, sequencing efforts were focused on a single target pathogen, such as influenza viruses, whereas in the present study our approach aims to simultaneously detect a broad panel of relevant respiratory viruses in a single assay. It has also been applied for the rapid detection of antimicrobial resistance (AMR) genes directly from clinical specimens (Serpa et al., 2022), highlighting an additional area where the MiFi^®^ software could expand by designing dedicated e-probes for high-priority resistance determinants. This approach allows clinicians to recommend targeted treatments, avoid unnecessary therapies, and detect unexpected or fastidious organisms that would likely be missed by conventional culture-based or PCR diagnostics (Chapman et al., 2023; Charalampous et al., 2024). In these settings, NGS has proven particularly valuable for resolving cases of unknown etiology and guiding precision medicine strategies. However, its adoption in low-resource settings is often limited by extensive data analysis requirements that demand trained bioinformaticians (Miah et al., 2023). The MiFi^®^ software overcomes this barrier by delivering results within minutes of data upload, eliminating the need for specialized expertise and making metagenomic diagnostics more accessible for hospitals and diagnostic centers (Espindola and Cardwell, 2021).

Instances where MiFi^®^ detected viruses in PCR-negative samples raise two possibilities: false negatives by PCR, due to low viral load, primer mismatches, or suboptimal amplification conditions, or artifacts inherent to HTS workflows, such as carryover between multiplexed samples (Nascimento et al., 2025). The presence of substantial genome coverage and statistically significant *p*-values supports the likelihood that some of these cases represent true positives. Nevertheless, additional measures, such as including blanks or “alien controls” between diagnostic samples, are recommended to minimize the risk of cross-contamination (Rong et al., 2023).

The implications of this work extend to both public and animal health. Rapid and accurate detection of respiratory pathogens using MiFi^®^ can guide clinical decision-making, prevent unnecessary treatments, support outbreak control measures, and strengthen epidemiological surveillance systems.

This study has limitations, including the small sample size for certain viruses, such as IBV, which restricts the statistical robustness of sensitivity estimates. Furthermore, only five of the nine targets were validated in real samples, and the performance of e-probes is inherently linked to the quality and comprehensiveness of publicly available sequence data.

Future work will focus on expanding *in-vivo* validation to all target pathogens with larger and more diverse sample sets, as well as broadening our pathogen library through the design of additional e-probes. We also aim to adapt and deploy this workflow beyond controlled laboratory settings, bringing it to locations where rapid diagnosis is most needed. By integrating portable extraction and sequencing solutions with the MiFi^®^ software, the protocol could be implemented directly at community clinics, veterinary field stations, or outbreak sites, enabling near real-time, accurate pathogen detection at the point of sample collection and strengthening response capacity in both human and animal health sectors.

In summary, this work advances current diagnostic paradigms by demonstrating that e-probe–based metagenomic screening can be applied simultaneously to a broad panel of clinically and veterinary relevant respiratory viruses in a single untargeted assay, maintaining high analytical specificity and competitive sensitivity. By integrating streamlined bioinformatics through the MiFi^®^ software with portable sequencing technologies, our approach bridges the gap between high-throughput sequencing and routine diagnostics, extending the benefits of metagenomics beyond specialized laboratories. This capability not only supports rapid, evidence-based clinical decision-making but also strengthens real-time surveillance and outbreak response capacity across both human and animal health sectors.

## 5 Conclusion

MiFi^®^ detected a broad panel of human and veterinary respiratory viruses from raw HTS data with high analytical specificity and competitive sensitivity. Across 83 *in-silico* datasets, curated e-probes achieved 100% specificity and limits of detection as low as 0.0010% of total reads (equivalent to 10 reads per 10^6^) for some targets, and in clinical validation with ONT datasets, MiFi^®^ matched PCR performance for HRSV, IBV, IAV, BRSV, and CDV. Taken together, these results indicate that MiFi^®^ can lower practical barriers to routine metagenomic diagnostics by multiplexing many pathogens at once and reducing the need for specialized bioinformatics, thereby enabling faster triage, surveillance, and outbreak response in both clinical and veterinary settings.

## Data Availability

The datasets analyzed in this study are available in the NCBI database (https://www.ncbi.nlm.nih.gov/), with accession numbers provided in Supplementary Tables 1–3.

## Footnotes

1
http://bioinfo.okstate.edu

2
http://mifi.tech

3
http://apsnet.org/DAVN/Tools/Calculators/Pages/default.aspx

## References

[B1] Bocsanczy A. M. Espindola A. S. Cardwell K. Norman D. J. (2023). Development and validation of E-Probes with the MiFi system for detection of *Ralstonia solanacearum* species complex in blueberries. *PhytoFrontiers*™ 3 137–147. 10.1094/PHYTOFR-04-22-0043-FI

[B2] Cane J. Sanderson N. Barnett S. Vaughan A. Pott M. Kapel N. (2024). Nanopore sequencing of influenza A and B in oxfordshire and the United Kingdom, 2022–23. *J. Infect.* 88:106164. 10.1016/j.jinf.2024.106164 38692359 PMC11101610

[B3] Chapman R. Jones L. D’Angelo A. Suliman A. Anwar M. Bagby S. (2023). Nanopore-based metagenomic sequencing in respiratory tract infection: A developing diagnostic platform. *Lung* 201 171–179. 10.1007/s00408-023-00612-y 37009923 PMC10067523

[B4] Charalampous T. Alcolea-Medina A. Snell L. B. Alder C. Tan M. Williams T. G. (2024). Routine metagenomics service for ICU patients with respiratory infection. *Am. J. Respir. Crit. Care Med.* 209 164–174. 10.1164/rccm.202305-0901OC 37938162 PMC10806431

[B5] Chiu C. Y. Miller S. A. (2019). Clinical metagenomics. *Nat. Rev. Genet.* 20 341–355. 10.1038/s41576-019-0113-7 30918369 PMC6858796

[B6] Dabbagh A. (2018). Progress toward regional measles elimination—worldwide, 2000–2017. *MMWR. Morbidity Mortal. Weekly Rep.* 67 1323–1329. 10.15585/mmwr.mm6747a6 30496160 PMC6276384

[B7] Dang T. Wang H. Espindola A. S. Habiger J. Vidalakis G. Cardwell K. (2023). Development and statistical validation of e-probe diagnostic nucleic acid analysis (EDNA) assays for the detection of citrus pathogens from raw high-throughput sequencing data. *PhytoFrontiers*™ 3 113–123. 10.1094/PHYTOFR-05-22-0047-FI

[B8] Espindola A. S. (2024). Simulated high throughput sequencing datasets: A crucial tool for validating bioinformatic pathogen detection pipelines. *Biology* 13:700. 10.3390/biology13090700 39336128 PMC11428249

[B9] Espindola A. S. Cardwell K. F. (2021). Microbe finder (Mifi§): Implementation of an interactive pathogen detection tool in metagenomic sequence data. *Plants* 10:250. 10.3390/plants10020250 33525397 PMC7912148

[B10] Espindola A. S. Schneider W. Cardwell K. F. Carrillo Y. Hoyt P. R. Marek S. M. (2018). Inferring the presence of aflatoxin-producing *Aspergillus flavus* strains using RNA sequencing and electronic probes as a transcriptomic screening tool. *PLoS One* 13:e0198575. 10.1371/JOURNAL.PONE.0198575 30325975 PMC6191106

[B11] Groth-Helms D. Dennis G. Turecheck W. Yasuhara-Bell J. Eads A. Cardwell K. (2025). *DAVN Statistics Toolbox.* St. Paul, MN: APSNET.

[B12] Leinonen R. Sugawara H. Shumway M. Collaboration I. N. S. D. (2010). The sequence read archive. *Nucleic Acids Res.* 39 D19–D21. 10.1093/nar/gkq1019 21062823 PMC3013647

[B13] Mackay I. M. Arden K. E. Nitsche A. (2002). Real-time PCR in virology. *Nucleic Acids Res.* 30 1292–1305. 10.1093/nar/30.6.1292 11884626 PMC101343

[B14] Miah M. Hossain M. E. Hasan R. Alam M. S. Puspo J. A. Hasan M. M. (2023). Culture-independent workflow for nanopore MinION-based sequencing of influenza A virus. *Microbiol. Spect.* 11:e04946-22. 10.1128/spectrum.04946-22 37212605 PMC10269883

[B15] Minor N. R. Ramuta M. D. Stauss M. R. Harwood O. E. Brakefield S. F. Alberts A. (2023). Metagenomic sequencing detects human respiratory and enteric viruses in air samples collected from congregate settings. *Sci. Rep.* 13:21398. 10.1038/s41598-023-48352-6 38049453 PMC10696062

[B16] Narayanan S. Espindola A. S. Malayer J. Cardwell K. Ramachandran A. (2023). Development and evaluation of Microbe Finder (MiFi)§: A novel in silico diagnostic platform for pathogen detection from metagenomic data. *J. Med. Microbiol.* 72:001720. 10.1099/jmm.0.001720 37345698

[B17] Nascimento D. Bodaghi S. Wang H. Ribeiro-Junior M. Campos R. Dang T. (2025). Development and validation of a suite of E-Probes for Electronic Diagnostic Nucleic Acid Analysis (EDNA) for 20 graft-transmissible pathogens of citrus using MiFi^®^ and blind ring testing among novice users. *PhytoFrontiers* 5 243–253. 10.1094/PHYTOFR-12-24-0140-FI

[B18] Pasha A. Espindola A. S. Ziebell H. Ochoa-Corona F. M. (2024). Highly curated and reliable e-probes for detection of viral pathogens in unassembled high-throughput sequencing datasets from hops. *PhytoFrontiers*™ 5 165–173. 10.1094/PHYTOFR-09-24-0106-FI

[B19] Patel M. K. (2020). Progress toward regional measles elimination—worldwide, 2000–2019. *MMWR. Morbidity Mortal. Weekly Rep.* 69 1700–1705. 10.15585/mmwr.mm6945a6 33180759 PMC7660667

[B20] Peña-Zúñiga L. Espindola A. Hagen D. Ali A. Ochoa-Corona F. (2025). Assessment of viral limit of detection in spiked, unassembled high-throughput sequencing datasets. *PhytoFrontiers*™ 5 264–271. 10.1094/PHYTOFR-11-24-0121-FI

[B21] Proaño-Cuenca F. Carrera-López D. Zeller K. Espindola A. S. Cardwell K. (2025). Integrating in silico and in vitro approaches for detecting Coniothyrium glycines in high-throughput sequencing (HTS) datasets using EDNA-MiFi. *PhytoFrontiers*™ 5 254–263. 10.1094/PHYTOFR-11-24-0121-FI

[B22] Proaño-Cuenca F. Espindola A. S. Garzon C. D. (2023). Detection of phytophthora, pythium, globisporangium, hyaloperonospora, and plasmopara species in high-throughput sequencing data by in silico and in vitro analysis using Microbe Finder (MiFi). *PhytoFrontiers*™ 3 124–136. 10.1094/PHYTOFR-04-22-0039-FI

[B23] Ribeiro-Junior M. R. Espindola A. Nascimento D. M. Da Silva F. B. Krause-Sakate R. Ochoa-Corona F. M. (2025). An attempt toward the global screening of soybean viruses using EDNA-MiFi-Based electronic probes. *PhytoFrontiers*™ 5 236–242. 10.1094/PHYTOFR-12-24-0141-FI

[B24] Rong W. Rollin J. Hanafi M. Roux N. Massart S. (2023). Validation of high-throughput sequencing as virus indexing test for musa germplasm: Performance criteria evaluation and contamination monitoring using an alien control. *PhytoFrontiers*™ 3 91–102. 10.1094/PHYTOFR-03-22-0030-FI

[B25] Roy A. Shao J. Espindola A. S. Ramos Lopez D. Otero-Colina G. Rivera Y. (2025). Detection and in vivo validation of dichorhavirus e-probes in meta-transcriptomic data via Microbe Finder (MiFi§) discovers a novel host and a possible new strain of orchid fleck virus. *Viruses* 17:441. 10.3390/v17030441 40143368 PMC11946451

[B26] Serpa P. H. Deng X. Abdelghany M. Crawford E. Malcolm K. Caldera S. (2022). Metagenomic prediction of antimicrobial resistance in critically ill patients with lower respiratory tract infections. *Genome Med.* 14:74. 10.1186/s13073-022-01072-4 35818068 PMC9275031

[B27] Stobbe A. H. Daniels J. Espindola A. S. Verma R. Melcher U. Ochoa-Corona F. (2013). E-probe Diagnostic Nucleic acid Analysis (EDNA): A theoretical approach for handling of next generation sequencing data for diagnostics. *J. Microbiol. Methods* 94 356–366. 10.1016/j.mimet.2013.07.002 23867249

[B28] Wang X. Stelzer-Braid S. Scotch M. Rawlinson W. D. (2022). Detection of respiratory viruses directly from clinical samples using next-generation sequencing: A literature review of recent advances and potential for routine clinical use. *Rev. Med. Virol.* 32:e2375. 10.1002/rmv.2375 35775736 PMC9539958

[B29] Williams T. G. Snell L. B. Alder C. Charalampous T. Alcolea-Medina A. Sehmi J. K. (2023). Feasibility and clinical utility of local rapid Nanopore influenza A virus whole genome sequencing for integrated outbreak management, genotypic resistance detection and timely surveillance. *Microb. Genom.* 9:mgen001083. 10.1099/mgen.0.001083 37590039 PMC10483427

[B30] World Health Organization (2019). *Measles and Rubella Surveillance Data.* Geneva: WHO.

