# Supplementary material for: Rapid detection of human and animal respiratory viruses using Microbe Finder (MiFi®)
Source: Front Microbiol. 2026 Feb 25;17:1743643. doi: 10.3389/fmicb.2026.1743643 (PMC12999926; doi:10.3389/fmicb.2026.1743643)
Supplement: Supplementary file 1 [file Table_1.pdf]

Supplementary Table 1 - Inclusivity/exclusivity panels and genomic data for selected target viruses and near-neighbors.

Common name (acronym)	Viral species	Inclusivity Panel	Exclusivity Panel
		**Human viruses**	
Human parainfluenza virus 4 (HPIV4)	*Orthorubulavirus hominis*	All *Orthorubulavirus hominis* (HPIV4a/b) [NCBI:txid2560526]	All *Morbillivirus*, all *Orthorubulavirus* (except HPIV4a/b), all *Orthopneumovirus*, all *Rhinovirus*, and all *Coronavirus*
Human respiratory syncytial virus (HRSV)	*Orthopneumovirus hominis*	All *Orthopneumovirus hominis* (HRSV) [NCBI:txid11250]	All *Morbillivirus*, all *Orthorubulavirus*, all *Orthopneumovirus* (except HRSV), all *Rhinovirus*, and all *Coronavirus*
Influenza A virus (IAV)	*Alphainfluenzavirus influenzae*	All *Influenza A virus* (IAV) [NCBI:txid11320]	All *Alphainfluenzavirus* (except IAV), all *Betainfluenzavirus*, all *Deltainfluenzavirus*, all *Gammainfluenzavirus*, all *Isavirus*, all *Mykissvirus*, all *Quaranjavirus*, all *Sardinovirus*, all *Thogotovirus*, and all unclassified *Orthomyxoviridae*
Influenza B virus (IBV)	*Betainfluenzavirus influenzae*	All *Influenza B virus* (IBV) [NCBI:txid11520]	All *Alphainfluenzavirus* (except IBV), all *Betainfluenzavirus*, all *Deltainfluenzavirus*, all *Gammainfluenzavirus*, all *Isavirus*, all *Mykissvirus*, all *Quaranjavirus*, all *Sardinovirus*, all *Thogotovirus*, and all unclassified *Orthomyxoviridae*
Measles virus (MeV)	*Morbillivirus hominis*	All *Morbillivirus hominis* (MeV) [NCBI:txid11234]	All *Morbillivirus* (except MeV), all *Orthorubulavirus*, all *Orthopneumovirus*, all *Rhinovirus*, and all *Coronavirus*
Measles virus B3 (MeV-B3)	*Morbillivirus hominis*	All *Morbillivirus hominis* (MeV-B3) [NCBI:txid658048]	All *Morbillivirus* (except MeV-B3), all *Orthorubulavirus*, all *Orthopneumovirus*, all *Rhinovirus*, and all *Coronavirus*
Measles virus D8 (MeV-D8)	*Morbillivirus hominis*	All *Morbillivirus hominis* (MeV-D8) [NCBI:txid170528]	All *Morbillivirus* (except MeV-D8), all *Orthorubulavirus*, all *Orthopneumovirus*, all *Rhinovirus*, and all *Coronavirus*
Measles virus Edwt (MeV-Edwt)	*Morbillivirus hominis*	Edmonston wild-type strain, GenBank:AF266288 [NCBI:txid11235]	All *Morbillivirus* (MeV) strains except Edmonston
Measles virus Mor (MeV-Mor)	*Morbillivirus hominis*	Moraten strain, GenBank:AF266287 [NCBI:txid132484]	MeV vaccine strains: Zagreb, A1KC, Schwarz, Rubeovax, and Changchun-47

Mumps virus (MuV)	*Orthorubulavirus parotitidis*	All *Orthorubulavirus parotitidis* (MuV) [NCBI:txid2560602]	All *Morbillivirus*, all *Orthorubulavirus*, all *Orthopneumovirus* (except MuV), all *Rhinovirus*, and all *Coronavirus*
**Animal viruses**			
Bovine respiratory syncytial virus (BRSV)	*Orthopneumovirus bovis*	All *Orthopneumovirus bovis* (BRSV) [NCBI:txid3136119]	All *Morbillivirus*, all *Orthorubulavirus*, all *Orthopneumovirus* (except BRSV), all *Rhinovirus*, and all *Coronavirus*
Canine distemper virus (CDV)	*Morbillivirus canis*	All *Morbillivirus canis* (CDV) [NCBI:txid3139435]	All *Morbillivirus* (except CDV), all *Orthorubulavirus*, all *Orthopneumovirus*, all *Rhinovirus*, and all *Coronavirus*
Feline morbillivirus (FeMV)	*Morbillivirus felis*	All *Morbillivirus felis* (FeMV) [NCBI:txid1170234]	All *Morbillivirus* (except FeMV), all *Orthorubulavirus*, all *Orthopneumovirus*, all *Rhinovirus*, and all *Coronavirus*
