# Supplementary material for: Rapid detection of human and animal respiratory viruses using Microbe Finder (MiFi®)
Source: Front Microbiol. 2026 Feb 25;17:1743643. doi: 10.3389/fmicb.2026.1743643 (PMC12999926; doi:10.3389/fmicb.2026.1743643)
Supplement: Supplementary file 3 [file Table_3.pdf]

Supplementary Table 3 - Reference genomes used for *in-silico* sensitivity testing with mock datasets.

**Target pathogen**	**Acronym**	**Isolate**	**NCBI accession number(s)**
		**Human viruses**	
Human parainfluenza virus 4	HPIV4	M-25	AB543336
Human respiratory syncytial virus	HRSV	A2	M74568
Influenza A virus	IAV	A/Puerto Rico/8/1934 (H1N1)	NC_002023.1, NC_002050.1, NC_002022.1, NC_002017.1, NC_002019.1, NC_002021.1, NC_002016.1, NC_002018.1
Influenza B virus	IBV	B/Lee/1940	NC_002205.1, NC_002204.1, NC_002226.1, NC_002198.1, NC_002199.1, NC_002200.1, NC_002201.1, NC_002202.1
Measles virus	MeV	Ichinose-B95a	AB016162
Measles virus B3	MeV-B3	MVs/Tours.FRA/49.18[B3] (MIBE)	MN893225
Measles virus D8	MeV-D8	MVi/Texas.USA/4.07	JN635407
Measles virus Edwt	MeV-Edwt	Edmonston wild type	AF266288
Measles virus Mor	MeV-Mor	Moraten	AF266287
Mumps virus	MuV	Miyahara	AB040874
		**Animal viruses**	
Bovine respiratory syncytial virus	BRSV	ATCC 51908	AF295543
Canine distemper virus	CDV	Onderstpoort	AF014953
Feline morbillivirus	FeMV	761U	JQ411014
